# Neurological Diseases With Autism Spectrum Disorder: Role of ASD Risk Genes

**DOI:** 10.3389/fnins.2019.00349

**Published:** 2019-04-11

**Authors:** Juan Xiong, Shimeng Chen, Nan Pang, Xiaolu Deng, Lifen Yang, Fang He, Liwen Wu, Chen Chen, Fei Yin, Jing Peng

**Affiliations:** ^1^Departmen of Pediatrics, Xiangya Hospital, Central South University, Changsha, China; ^2^Hunan Intellectual and Developmental Disabilities Research Center, Changsha, China

**Keywords:** autism risk gene, autism spectrum disorder, gene function, neurological diseases, risk factors

## Abstract

Autism spectrum disorder (ASD) is frequently comorbid with other neurological disorders such as intellectual disability (ID) or global development delay (GDD) and epilepsy. The pathogenesis of ASD is complex. So far, studies have identified more than 1000 ASD risk genes. Most of them were also reported to relate with other neurological diseases, and only several of them have been confirmed as pathogenic genes for autism. Little is known about the roles of these risk genes in neurological diseases with ASD. In the present study, we recruited a cohort of 158 neurological disorder probands with 163 variants of 48 ASD risk genes. Of these, 50 individuals (31.6%) were diagnosed with ASD. In the ASD patient subset, we identified several rarely reported candidate genes including *DOLK*, *USH2A*, and *HUWE1*. In a comparison of patients with neurological disorders with and without ASD, we found that ID/GDD was frequently comorbid with ASD whereas epilepsy was more common in the non-ASD group. Statistical analyses of all possible risk factors implicated that variants in synaptic genes, especially non-voltage-gated ion channel genes and in transcriptional and chromosome genes were related to ASD, but none of the investigated environmental factors was. Our results are useful for the future diagnosis and prognosis of patients with neurological disorders and emphasize the utility of genetic screening.

## Introduction

Autism spectrum disorder (ASD), a common neurodevelopmental disorder, is characterized by deficiency in social communication and interaction and restricted, repetitive behaviors (Lai et al., 2014). ASD is often associated with other neurological comorbidities such as epilepsy and intellectual disability (ID) (Yeargin-Allsopp et al., 2003; Sundelin et al., 2016). This phenomenon indicates a possible shared etiology between ASD and other neurological diseases. Recent studies have shown that neurological disorders such as epilepsy and ID are multifactorial and involve both genetic and environmental contributions (Chiurazzi and Pirozzi, 2016; Mazarati et al., 2017; Bozzi et al., 2018). The increasing prevalence of genetic testing has permitted the discovery of various relationships between multiple genes and various neurological disorders. ASD has 1007 risk genes as per the SFARI Gene database^1^, an evolving database of genes that are implicated in autism susceptibility. Few causative genes have been identified for ASD, including *MECP2* and *CHD8* (Li et al., 2017). Many other ASD-associated genes like *CDKL5*, *SCN1A* are also associated with epilepsy, ID, or other neurological syndromes (Weaving et al., 2004; Meng et al., 2015). Alternatively, several epidemiological studies have demonstrated the importance of environmental risk factors for ASD including advanced parental age; maternal smoking; pregnancy-related factors such as obesity and diabetes; and postnatal exposure to heavy metals, air pollutants, or other toxins (Modabbernia et al., 2017; Wu et al., 2017).

Genetic mutations in ASD risk genes have been detected in children with a variety of nervous system disorders, including epilepsy and ID. Yet, only a proportion of these patients present with autistic traits, even when mutations occur in syndromic ASD genes such as *TSC1* (Numis et al., 2011). Therefore, we examined possible molecular genetic and/or environmental factors driving phenotypic differences in these patients.

Here, we recruited 158 patients with neurological disorders and mutations in ASD risk genes in order to examine the contributions of genetic and environmental influences to the manifestation of ASD and neurological comorbidities.

## Materials and Methods

### Patients

We recruited 158 patients with nervous system disorders from the pediatric department of Xiangya Hospital at Central South University from 2014 to 2018. Their initial diagnoses included epilepsy, ID or global developmental delay (GDD), tuberous sclerosis complex, Rett syndrome, or other neurological disorder. All subjects have one or more pathogenic/likely pathogenic/uncertain significant variants in ASD risk genes. ASD risk genes were identified according to the SFARI Gene database^2^. We then adopted “genotype-first approach” to confirm the final diagnoses of patients (Stessman et al., 2014). All diagnoses were supported by the genetic results. Ethical approval for this study was obtained from Xiangya Hospital Ethics Committee in accordance with Helsinki declaration, and informed consent was obtained from subjects and the parents or guardians of patients.

Follow-up was performed from the date of the genetic diagnosis to the date of a diagnosis of ASD or until June 1, 2018 (whichever occurred first) unless patients withdrew informed consent or died. Patients were evaluated for ASD in accordance with the DSM-V criteria by two experienced pediatric neurological doctors: (1) persistent deficits in social communication and social interaction; (2) restricted, repetitive patterns of behavior, interests, or activities; (3) symptoms present in the early developmental period; (4) symptoms caused clinically significant impairment in social, occupational, or other critical areas of functioning; and (5) disturbances were not better explained by ID or GDD. The diagnostic procedure also included the assessment using a series of tools for neurological examination, mental status examination. Then, we divided the cohort into two groups: patients with ASD group (ASD group) and patients without ASD group (non-ASD group). Variant information for each gene and ASD environmental risk factors (i.e., parental age; maternal factors including obesity, diabetes, and caesarian section birth; and birth complications including hypoxia and intracranial hemorrhage, collected from a detailed patient history) were used to analyze differences between the two groups.

### Capture Strategy, Sequencing, and Validation

All patients were subjected to peripheral blood genetic testing. Forty-four patients underwent whole-exome sequencing (WES), 67 patients underwent gene panel testing including panels for epilepsy, tuberous sclerosis complex, and neurofibromatosis, and 47 patients underwent clinical WES gene panel testing. The methods for sequencing and genetic testing have been described in detail in a previous study (Arafat et al., 2017; Peng et al., 2018). Candidate variants were filtered as per the American College of Medical Genetics (ACMG) guidelines for the interpretation of sequence variants (Richards et al., 2008) and confirmed by Sanger sequencing in probands and their parents, if genetic samples were available. Segregation analyses were also performed on family members.

### Genetic Functional Grouping

The present study only included ASD risk genes confirmed by the SFARI Gene database. Many of these genes encode proteins related to synaptic formation, transcriptional regulation, and chromatin remodeling (De Rubeis et al., 2014). Combined with related literature and SFARI Gene database, mutated genes in this study were grouped into three categories: (1) synaptic genes: genes involved in synaptic function such as transmission, vesicle recycling, and synaptic plasticity (Auerbach et al., 2011; Santini et al., 2013); (2) transcriptional and chromatin genes: chromatin regulators and transcription factors that are essential for developmental processes, including ATP-dependent chromatin remodelers, chromatin modifiers, and other chromatin remodelers (Jenuwein and Allis, 2001; Ho and Crabtree, 2010; Torigoe et al., 2013); and (3) genes with other functions including metabolism-related genes (Helander et al., 2013; Rumsey et al., 2014). In order to identify the role of voltage-gated ion channels in patients with ASD, synaptic genes were further divided into two groups: genes encoding voltage-gated ion channels and genes encoding non-voltage-gated ion channels.

### Statistics

Significant differences in initial diagnoses between the ASD and non-ASD groups were evaluated using Fisher’s exact tests. Significant differences in the presence of ASD were evaluated for each gene function group, each mutation type, and each possible environmental risk factor using Fisher’s exact tests for R × C matrix. The threshold for significance was *p* < 0.05. To control for multiple comparisons, we used the Benjamini–Yekutieli approach, which allows for dependent data structures, setting a false discovery rate (FDR) of 0.05.

## Results

### Segregation Analysis

This cohort included 158 cases of neurological disorders. Some of the patients were siblings, and they were patient B0019 and A0251, M003 and M0346 as well as M0334 and M0335. In total, 131 of the cases had undergone segregation analyses while others did not due to lack of the parental genetic samples. Among these 131 cases, 93 had *de novo* mutations. Six cases were detected with compound heterozygous mutations which were inherited from their healthy parents. Notably, proband M0332 had a *de* *novo* mutation and heterozygous mutation inherited from his healthy mother. Eleven probands had heterozygous mutations that were also detected in their affected parents. Moreover, 12 male children had a hemizygous variant of X-linked recessive genes derived from their normal mothers. Seven patients shared a heterozygous *SCN1A* (M0343, M0344, P0274, M0352, M0353, M0355) or *SCN8A* (M0358) mutation with their healthy parents respectively, which can be explained as incomplete penetrance. Besides, patient M0046 who had an *STXBP1* mutation inherited from the healthy mother due to mosaicism as was reported in a previous research (Peng et al., 2018) (Supplementary Table S1).

### Patient Phenotyping

Two subjects died during the follow-up period. Accordingly, the analysis included 156 patients with a mean age of 57.24 ± 32.37 months. The male to female ratio was 1.08 (81:75). Initial diagnoses before genetic testing included epilepsy (58 patients, 37.2%), epileptic encephalopathy (38 patients, 24.4%), ID/GDD (30 patients, 19.2%), both ID/GDD and epilepsy (IDE) (10 patients, 6.4%), febrile seizures, febrile seizures plus, or complex febrile seizures (7 patients, 4.5%), tuberous sclerosis (4 patients, 2.6%), and other nervous system diseases (9 patients, 5.8%) (Supplementary Table S1).

By June 1st, 2018, 50 (31.6%) of patients had been diagnosed as ASD or co-comorbid ASD. The ratio of sex in patients with ASD was 1:1. The average age at diagnosis of ASD was 55.58 ± 33.997 months (Supplementary Table S1). With regard to comorbid diseases with ASD, ID/GDD was more prevalent in the ASD group than in the non-ASD group (50.0% vs. 5.7%, *p* = 9.37 × 10^-11^). In contrast, epilepsy and epileptic encephalopathy were less present in the ASD group than in the non-ASD group (34.0% vs. 74.5%, *p* = 2.76 × 10^-4^) (Table 1).

**Table 1:** Initial diagnosis of patients with neurological diseases with or without comorbid autism spectrum disorder (ASD).

Initial diagnosis	ASD group	Non-ASD group	Significance	Odds Ratio
ID	25	6	***p* = 9.37 × 10^-11^**	16.667
No ID	25	100		
Epilepsy	17	79	***p* = 2.76 × 10^-4^**	0.253
No epilepsy	33	27		


*Statistically significant findings are bolded. ID, intellectual disability.*

### Patient Genotyping

We detected 163 variants in 48 ASD risk genes. Only one mutated gene was identified as a pathogenic or likely pathogenic or uncertain significant gene in each subject. 157 variants in 44 ASD risk genes were likely pathogenic or pathogenic, and 6 variants of 4 genes are uncertain significant. 3/50 ASD group patients and 4/108 probands in the non-ASD group had more than one variants while the rest had only one variant each. The most frequently mutated gene was *SCN1A* (56, 35.4%), followed by *CDKL5* (16, 10.1%), *MECP2* (7, 4.4%), *PCDH19* (4, 2.5%), *ATP1A3* (4, 2.5%), *NF1* (4, 2.5%), *SCN8A* (4, 2.5%), *TSC2* (4, 2.5%), *SCN2A* (3, 1.9%), *TSC1* (3, 1.9%), *SMARCA2* (3, 1.9%), and *STXBP1* (3, 1.9%) (Supplementary Table S1). Only 50 patients with 54 variants of 29 genes had autistic characteristics; these variants included 27 missense mutations, 9 nonsense mutations, 3 splice variants, 11 deletions, 2 duplications, and 2 insertions. The most common genes in this group were *CDKL5* (8, 16%), *MECP2* (7, 14%), *SMARCA2* (3, 6%), *SYNGAP1* (2, 4%), *SLC6A1* (2, 4%), and *UBE3A* (2, 4%) (Supplementary Table S1). It is noteworthy that variants of *USH2A*, *HUWE1*, and *DOLK* were detected in patients with ASD, which has been rarely reported.

### Gene Functions and Mutation Types in the ASD and Non-ASD Groups

Gene functional classifications and the numbers of genes in each group are shown in Tables 2, 3. A comparison of the ASD and non-ASD groups revealed significant differences among the 3 gene function groups (*p* = 2.63 × 10^-7^, FDR = 9.21 × 10^-7^). There was a higher proportion of patients with transcriptional and chromosomal gene mutations (Group b) in the ASD group (25/50, 50.0%) than in the non-ASD group (15/106, 14.2%) (*p* = 2.00 × 10^-6^, FDR = 3.5 × 10^-6^), while the contrary was observed for mutations in synaptic genes (Group a) (21/50, 42% vs. 89/106, 84.0%, respectively; *p* = 8.14 × 10^-8^, FDR = 5.70 × 10^-7^). Furthermore, the number of ASD patients between Group a and b had significant differences (Group a vs. Group b, *p* = 3.42 × 10^-7^, FDR = 7.98 × 10^-7^), but Group a and c(*p* = 1.91 × 10^-2^, FDR = 2.23 × 10^-2^), Group b and c (*p* = 1, FDR = 1) didn’t. Additionally, there was a higher proportion of ASD patients with mutations in non-voltage-gated ion channel genes than voltage-gated ion channel genes (67.4% vs. 23.8%, *p* = 2.56 × 10^-4^, FDR = 3.58 × 10^-4^) (Table 4).

**Table 2:** Gene functional classifications by group.

Genotype grouping	ASD group	Non-ASD group
Synaptic genes	*ARHGEF9*(2), *SCN1A*(2), *CACNA1A*(1), *SCN2A*(1), *CC2D1A*(1), *SCN8A*(1), *SLC6A1*(2), *SLC6A8*(1), *SYNGAP1*(2), *TSC1*(2), *TSC2*(1), *USH2A*(1), *DYRK1A*(1), *UBE3A*(2), *FRMPD4*(1)	*ATP1A3*(4), *GRIN2B*(1), *TSC1*(1), *KCNQ3*(1), *SCN1A*(54), *TSC2*(3), *SCN2A*(1), *SCN8A*(2), *SCN9A*(2), *SLC30A3*(1), *STXBP1*(3), *NF1*(4), *UBE3A*(1), *POMT1*(3), *ITPR1*(1), *PCDH19*(4), *RELN*(1), *PTEN*(1), *CTNNB1*(1)
Transcriptional and chromatin genes	*ARX*(1), *FOXG1*(1), *MECP2*(7), *CDKL5*(8), *MEF2C*(1), *HCFC1*(1), *HUWE1*(1), *CHD8*(1), *SMARCA2*(3), *ASXL3*(1)	*CDKL5*(8), *AFF2*(1), *DEAF1*(2), *ATRX*(1), *CHD2*(3)
Others	*DOLK*(1), *OCRL*(1), *SGSH*(1), *WDFY3*(1)	*OCRL*(1), *SPAST*(1)


*ASD, autism spectrum disorder. Numbers indicate the numbers of patients with mutations in each gene.*

**Table 3:** Synaptic gene subgroups.

Ion channel- related genes	*CACNA1A*, *SCN1A*, *SCN2A*, *SCN8A*, *KCNQ3*, *SCN9A*

Non-ion channel-related genes	*ARHGEF9*, *CC2D1A*, *SLC6A1*, *SYNGAP1*, *TSC1*, *TSC2*, *USH2A*, *DYRK1A*, *UBE3A*, *FRMPD4*, *GRIN2B*, *SLC30A3*, *STXBP1*, *NF1*, *POMT1*, *PCDH19*, *RELN*, *PTEN*, *CTNNB1, ATP1A3*, *ITPR1*, *SLC6A8*


**Table 4:** Gene functions of neurological diseases patients with or without autism spectrum disorder (ASD).

Gene functions	ASD group	Non-ASD group	Significance	Odds Ratio	False Discovery Rate
Group a	21	89	***p* = 2.63 × 10^-7^**	–	**9.21 × 10^-7^**
Group b	25	15			
Group c	4	2			
Group a	21	89	***p* = 8.14 × 10^-8^**	0.138	**5.70 × 10^-7^**
Group b and Group c	29	17			
Group b	25	15	***p* = 2.00 × 10^-6^**	6.067	**3.5 × 10^-6^**
Group a and Group c	25	91			
					
Group a	21	89	***p* = 3.42 × 10^-7^**	0.142	**7.98 × 10^-7^**
Group b	25	15			
Group a	21	89	***p* = 1.91 × 10^-2^**	0.118	**2.23 × 10^-2^**
Group c	4	2			
Group b	25	15	*p* = 1.000	0.833	1
Group c	4	2			
Genes encoding voltage-gated ion channels	5	60	***p* = 2.56 × 10^-4^**	0.151	**3.58 × 10^-4^**
Genes encoding non-voltage-gated ion channels	16	29			


*Statistically significant findings are bolded.*

Missense variants accounted for a substantial percentage of genetic mutations in both the ASD and non-ASD groups (50.0% vs. 52.3%), whereas nonsense variants were less frequent (16.7% vs. 14.4%). There is an uptrend of the nonsense mutations’ proportion of synaptic genes (21.4% vs. 13.0%) in the ASD group than in the non-ASD group, although no statistical differences have been found (Table 5).

**Table 5:** Mutation types of neurological diseases patients with or without autism spectrum disorder (ASD).

Mutation types	ASD group	Non-ASD group	Significance	Odds Ratio
**All patients**				
Missense	27	**58**	*p* = 0.676	-
Nonsense	9	16		
Deletion/duplication	13	22		
Insertion	2	2		
Splice	3	**13**		
Synaptic genes				
Missense	11	**47**	***p* = 0.274**	**0.620**
Non-missense	17	**45**		
				
Nonsense	6	**12**	***p* = 0.363**	**1.818**
Non-nonsense	22	**80**		


*Statistically significant findings are bolded.*

### Environmental Factors in Patients With or Without Comorbid ASD by Gene Mutation

We compared parental bearing age, maternal factors, delivery type, and birth complications between 50 patients with ASD and 100 patients without ASD (the related information of 8 patients in non-ASD group was not available) in our cohort. No significant between-group differences were detected (Table 6).

**Table 6:** Environmental risk factors in patients with neurological diseases with or without autism spectrum disorder (ASD).

Environmental risk factors	ASD group	Non-ASD group	Significance	Odds Ratio	False Discovery Rate
Delivery mode					
Cesarean section	23	43	*p* = 0.727	–	1.454
Natural labor	27	57			
Maternal factors					
Obesity	1	3	*p* = 1.000	–	1.0
No obesity	49	97			
Birth complications					
Hypoxia	2	9	*p* = 0.338	–	0.338
Normal	48	91			
Other birth complications	43	86	*p* = 1.000	–	1.0
Normal	7	14			
Paternal bearing age					
> 30 years old	19	39	*p* = 0.926	–	0.926
≤30 years old	24	52			
Maternal bearing age					
>30 years old	14	20	*p* = 0.262	–	0.262
≤30 years old	31	70			


## Discussion

Our data combined next-generation sequencing results with segregation analysis demonstrates that 163 variants in 48 ASD risk genes underlie at or associated to a subset of neurological disease in children, but autistic traits are not observed in every individual. On comparison between probands with or without ASD, some interesting phenomenon were found that not only patients’ phenotypes but also their mutated genes can be the risk factors.

### Unusual Sex Differences in Neurological Diseases in the ASD Group

Previous definitions of autism were very narrow. The newest DSM consolidated autistic disorder, Asperger’s disorder, and pervasive developmental disorder together into ASD. The prevalence of ASD has an obvious sex bias and is widely accepted to have a 4-fold incidence in men compared to that in women (Fombonne, 2009; Werling and Geschwind, 2013). Interestingly, the sex ratio of probands with ASD in our cohort was 1:1. There are several possibilities underlying this observation. Firstly, ID/GDD was a common comorbidity in our ASD cohort (29/50 patients), and previous studies have indicated that intelligence level significantly influences the sex ratio of autism, with a male-to-female ratio of 1.7:1 in patients with moderate-to-severe ID (Fombonne, 1999). Equally important, all subjects enrolled in the study had genetic mutations, which may have also contributed to the unusual sex ratio as women with autism have a higher rate of genetic variants than men with autism (Levy et al., 2011; Neale et al., 2012). Additionally, boys with externalizing behaviors including aggressive behavior, hyperactivity, and repetitive or restricted movements or interest (Hattier et al., 2011; Szatmari et al., 2012) are more frequently associated with ASD than girls with internalizing symptoms such as anxiety, depression, and other emotional manifestations (Bölte et al., 2011; Hattier et al., 2011; Mandy et al., 2012). Therefore, previously reported sex ratios might be offset by physician bias.

### ASD Risk Gene Mutations in Pediatric Neurological Disorder Patients

Several studies have described comorbid epilepsy, developmental disorders, psychiatric disorders, and other diseases in patients with autism (Simonoff et al., 2008; Hofvander et al., 2009; Mattila et al., 2010). With advancements in genetic testing, several genes have been identified as common to different neurological disorders. For example, mutations in *MECP2* have been detected in autism, ID, and epilepsy. A shared genetic basis for these disorders reflects the possibility of a common underlying mechanism. Additionally, overlap in the diagnostic criteria for these diseases may also explain a high frequency of comorbidity. A recent study showed that the prevalence of ASD has increased by 331% in 11 years (from 2000 to 2010) as a result of comorbidity with ID (Polyak et al., 2015).

In our cohort, ID and epilepsy were the two comorbid disorders most common with autism. A previous study reported that 70% of patients with autism had ID (Tuchman and Rapin, 2002), while the prevalence of autism with comorbid epilepsy has been estimated at 8–30% (Tuchman and Cuccaro, 2011). De Rubies and his colleagues also found that children with ID were more likely to develop ASD than those with epilepsy (De Rubeis et al., 2014). The similar conclusion could be obtained in our study, and we found that ID/GDD are more relevant with ASD than epilepsy. However, epilepsy coexisting with ID increases the likelihood of developing autistic features (Amiet et al., 2008). In our study, 6 of 10 patients with IDE (60%) were diagnosed with ASD. Since the sample size of the study is limited, we could not derive more useful conclusions on the relationships between other neurological diseases and ASD.

### Genes Contributing to a Possible Common Monogenic Mechanism in ASD

Previous studies have identified numerous genes associated with ASD, many of which encode proteins for synaptic transmission, synaptic formation, transcriptional regulation, and chromatin remodeling (De Rubeis et al., 2014). In order to better understand the possible mechanisms or pathways linking neurological disorders with ASD, we classified subjects into 3 groups according to the biological functions of mutated genes. The results illustrated that ASD risk genes with transcriptional and chromosome functions often associated with autistic features, whereas the opposite was observed for synaptic genes. We further concluded that ASD patients were more likely to have variants in non-ion channel-related genes than in ion channel-related genes. In a way, these general findings can assist clinicians in determining whether autistic characteristics require further monitoring and additionally benefit research.

Notably, 35.4% of patients in our cohort had *SCN1A* mutations, which have been previously associated with a spectrum of seizure disorders. Furthermore, all 56 of these patients were diagnosed with Dravet syndrome. ASD with *SCN1A* variants was first reported in Weiss et al. (2003), and subsequently identified in a large ASD cohort (Roak et al., 2011; De Rubeis et al., 2014). Animal studies have confirmed that *SCN1A* haploinsufficiency is associated with stereotyped behaviors and social interaction deficits (Han et al., 2012). Only 2 individuals with *SCN1A* variants had autistic traits in our study. In these subjects, seizures occurred in the first years of life, and the autistic behavior was observed at the ages of 5 and 9 years, respectively. Few studies have evaluated the relationship between Dravet syndrome and ASD; thus, it is difficult to predict whether the other 54 probands in our study with Dravet syndrome are likely to develop comorbid ASD. In contrast, 50% of our cases with *CDKL5* variants had ASD. This can be explained by the fact that *CDKL5* mutations associated to neurodevelopment disorders that have an obvious Rett-like phenotype (Fehr et al., 2015).

Remarkably, some genes such as *ARHGEF9*(2)*, SMARCA2*(4) were only detected in the ASD group but not in the non-ASD group while other genes like *ATP1A3*(4), *STXBP1*(3), *POMT1*(3), *CHD2*(3) were found in the non-ASD group. This phenomenon can be explained by the phenotype heterogeneity of the genes above; many of these genes were identified as candidate genes in ASD cohort studies but have also been implicated as causative genes for other neurological diseases. Therefore, it can be argued that ASD is not the usual clinical manifestation for these gene mutations. However, this could also be attributed to the imbalance of numbers in two groups. Studies involving much larger sample size are warranted.

### Variation in the Clinical Phenotypes of Patients With ASD Risk Genes

Although 158 probands in our cohort had mutations in autism risk genes, only 50 presented with autistic characteristics. It is known that interactions between environmental exposures and genes contribute to the incidence of autism. Therefore, we hypothesized that there would be genetic or environmental differences between the ASD and non-ASD groups, but only statistical differences exist between genetic factor and autistic traits in neurological disorder patients in our study.

Several studies have demonstrated that the genetic architecture of autism is complicated, and genes related to autism are pleiotropic (Wang et al., 2016; Guo et al., 2017; Li et al., 2017). Of 163 variants in 48 ASD risk genes detected in this cohort, only 54 variants in 29 genes were associated with autistic traits. Gene mutation types in both groups were similar, but the percentage of nonsense mutations was higher in the ASD group than in the non-ASD group, specifically for synaptic genes (21.4% vs. 13.0%). Sanders et al. (2012) demonstrated significant enrichment of nonsense mutations in patients with ASD but not in unaffected siblings. With the limitation of our study, we cannot draw a more accurate conclusion, but the tendency can be reflected by our results. In the literature, non-synonymous *de novo* single nucleotide variants in the form of splice-site and frameshift mutations also play an essential part in autism (Neale et al., 2012; Sanders et al., 2012); however, splice-site mutations and frameshift indels were not significantly associated with ASD in our cohort. This divergent finding might be attributable to the objective and methods of our research compared to other studies, as we focused on the role of ASD risk genes in pediatric neurological disease. Nevertheless, additional studies are needed to verify our results.

Epidemiological studies have identified various risk factors for ASD such as advanced paternal age (Lampi et al., 2013; Wu et al., 2017), birth complications, pregnancy-related factors, and postnatal exposure to toxins (Rossignol et al., 2014; Xu et al., 2014; Yoshimasu et al., 2014; Curran et al., 2015; Wang et al., 2016). In our study, there were no associations between potential environmental factors including parental age, cesarean section, birth complications, trauma, ischemia, hypoxia, and other exposures and ASD features. This may be related to the selection of subjects with known genetic mutations or a low number of subjects compared to that in larger previous studies and explain why our results are not in line with previous epidemiological findings.

### Outlook for ASD Risk Genes in Patients With Neurological Disorders

Autism spectrum disorder is frequently associated with other neurological disorders. An increasing number of causative genes for other neurological diseases are identified as candidate genes for ASD. Yet, it remains unclear if and when patients with these genes present with autistic characteristics and how they should be treated.

The present findings indicate that nearly 1/3 individuals with a nervous system disorder and a mutation in an ASD risk gene also develop comorbid ASD, especially in patients with ID. Further results indicated that gene functions and mutation types could also be used to predict ASD. For example, patients with variants in non-voltage gated ion channel related synaptic genes and transcriptional and chromosome genes were more likely to have comorbid ASD in our cohort. Thus, we recommend the use of genetic screening in patients with neurological disease and especially ID in order to identify patients that need to be monitored closely for the development of ASD.

## Ethics Statement

Ethical approval for this study was obtained from Xiangya Hospital Ethics Committee, and informed consent was obtained from subjects and from the parents or guardians of patients.

## Author Contributions

JX and SC played primary roles in study design, analyses, the literature review, and writing and revision of the manuscript. FH and CC collected the clinical data. NP and LW helped with the diagnosis of patients and the data analysis. XD and LY contributed to the qualitative coding and the writing of the manuscript. FY and JP conceptualized the study, led the literature search, and critically reviewed manuscript drafts for intellectual content.

## Conflict of Interest Statement

The authors declare that the research was conducted in the absence of any commercial or financial relationships that could be construed as a potential conflict of interest.

## Footnotes

1 https://gene.sfari.org/

2 https://gene.sfari.org/

## Supplementary Material

TABLE S1 Clinical features and genetic information of all subjects in the cohort.
